# Prognostic value of DNA flow cytometry in stomach cancer: a 5-year prospective study

**DOI:** 10.1038/sj.bjc.6690275

**Published:** 1999-04

**Authors:** K H Lee, J S Lee, J H Lee, S W Kim, C Suh, W K Kim, S-H Kim, Y I Min, B S Kim, K C Park, M S Lee, H S Sun

**Affiliations:** Department of MedicineGeneral SurgeryPreventive Medicine, University of Ulsan, College of Medicine, Asan Medical Center, 388-1 Poongnapdong, Songpa-ku, Seoul, 138-736, Korea; Department of Medicine, Catholic University Medical College, St. Mary Hospital, Seoul, 150-713, Korea

**Keywords:** stomach cancer, DNA flow cytometry, DNA ploidy, S-phase fraction, prognostic factor

## Abstract

The role of DNA flow cytometry in the prediction of prognosis for patients with stomach cancer remains to be defined. Thus we studied prospectively the role of DNA flow cytometry as a prognosis indicator in stomach cancer patients in a high-incidence area. Between November 1990 and December 1992, primary stomach cancer tissues were obtained from the surgical specimens from 217 patients (148 male, 69 female). DNA flow cytometric analyses of DNA ploidy and S-phase fraction were performed and the results were correlated with patient survival. The median age of the patients was 55 years (range 24–78). Aneuploid cell population was found in 114 of 217 samples (53%). Tumour S-phase fraction was obtained in 96 of 103 diploid tumours (93%) and 61 of 114 aneuploid tumours (54%). After median follow-up of 66.1 months, the patients with tumours with an S-phase fraction over 17% had significantly worse survival rates than patients with tumours with S-phase fractions of lower than 8% or 8–17% (45% vs 59% and 63% of patients surviving, *P* = 0.007). Tumour ploidy status did not correlate with patient survival. Multivariate analyses showed that the TNM stage remained the most important prognostic indicator. The tumour S-phase fraction was also an independent prognostic indicator (relative risk 2.300, 95% CI, 1.252–4.223). Tumour S-phase fraction obtained by DNA flow cytometry is an independent prognostic indicator for the survival of the patients with stomach cancer. © 1999 Cancer Research Campaign


*British Journal of Cancer* (1999) **79**(11/12), 1727–1735
© 1999 Cancer Research Campaign
Article no. bjoc.1998.0275

### Prognostic value of DNA flow cytometry in stomach cancer: a 5-year prospective study

**KH Lee[1], JS Lee[1], JH Lee[1], SW Kim[1], C Suh[1], WK Kim[1], S-H Kim[1], YI Min[1], BS Kim[2], KC Park[2], MS Lee[3] and HS Sun[4]**

Department of [1]Medicine, [2]General Surgery, and [3]Preventive Medicine, University of Ulsan, College of Medicine, Asan Medical Center, 388-1 Poongnapdong, Songpa-ku, Seoul, Korea 138-736; and [4]Department of Medicine, Catholic University Medical College, St. Mary Hospital, Seoul, Korea 150-713

**Summary** The role of DNA flow cytometry in the prediction of prognosis for patients with stomach cancer remains to be defined. Thus we studied prospectively the role of DNA flow cytometry as a prognosis indicator in stomach cancer patients in a high-incidence area. Between November 1990 and December 1992, primary stomach cancer tissues were obtained from the surgical specimens from 217 patients (148 male, 69 female). DNA flow cytometric analyses of DNA ploidy and S-phase fraction were performed and the results were correlated with patient survival. The median age of the patients was 55 years (range 24–78). Aneuploid cell population was found in 114 of 217 samples (53%). Tumour S-phase fraction was obtained in 96 of 103 diploid tumours (93%) and 61 of 114 aneuploid tumours (54%). After median follow-up of 66.1 months, the patients with tumours with an S-phase fraction over 17% had significantly worse survival rates than patients with tumours with S-phase fractions of lower than 8% or 8–17% (45% vs 59% and 63% of patients surviving, $P = 0.007$). Tumour ploidy status did not correlate with patient survival. Multivariate analyses showed that the TNM stage remained the most important prognostic indicator. The tumour S-phase fraction was also an independent prognostic indicator (relative risk 2.300, 95% CI, 1.252–4.223). Tumour S-phase fraction obtained by DNA flow cytometry is an independent prognostic indicator for the survival of the patients with stomach cancer.

**Keywords:** stomach cancer; DNA flow cytometry; DNA ploidy; S-phase fraction; prognostic factor

Although the mortality due to stomach cancer has decreased significantly over the last 5 decades in the USA and Western Europe, stomach cancer remains the leading cause of cancer mortality worldwide. Complete resection of the tumour is the treatment of choice, and an effective adjuvant treatment regimen has not been established (Hermans et al, 1993). The tumour stage is the most important factor in predicting patient survival after the surgery. We are in need of other biological markers that can predict patient survival and identify subsets of patients who might benefit from different therapeutic approaches.

DNA flow cytometry is a quantitative measure of DNA content (ploidy) and proliferative activity (S-phase fraction, SPF) of a tumour, and is hypothetically likely to give information regarding the subsequent clinical course of a patient with that particular tumour (Merkel et al, 1987). The measurement of tumour ploidy and tumour cell proliferation by DNA flow cytometry has been performed on a variety of human tumours in the past and shown to correlate with the prognosis of patients in several types of tumours, including colon and breast cancers (Look et al, 1988; Sigurdsson et al, 1990; Haffty et al, 1992; Bauer et al, 1993). Tumour SPF showed clear correlations with risk of recurrence and mortality for patients with both node-negative and node-positive breast cancer. Tumour ploidy of breast cancer also showed correlation with the prognosis of patients, although the magnitude of the difference was small (Hedley et al, 1993). Recent consensus review, however, did not recommend DNA flow cytometry for the routine management of colorectal and breast cancer, due to the lack of sufficient data showing the independent prognostic value of DNA flow cytometry (Bast et al, 1996). In stomach cancer, there have been numerous studies correlating DNA flow cytometry with patient prognosis. Several studies showed that patients with stomach cancer with an aneuploid tumour cell population or with a higher proliferative activity had worse prognoses (Tosi et al, 1988; Bronzo et al, 1989; Korenaga et al, 1989; Nanus et al, 1989; Wyatt et al, 1989; Yonemura et al, 1990; Baretton et al, 1991; Kimura and Yonemura, 1991; Johnson Jr et al, 1993; Kakeji et al, 1993; Rugge et al, 1994; Yonemura et al, 1994; D'Agnano et al, 1995; Flyger et al, 1995; Ikeguchi et al, 1995; Sakusabe et al, 1996; Victorzon et al, 1996). Other studies failed to show such correlation (Sasaki et al, 1989; Filipe et al, 1991; Sarbia et al, 1996). Most of these studies were performed in a retrospective manner using archived, paraffin-embedded tissues.

To ascertain the role of DNA flow cytometry as a prognostic indicator for patients with stomach cancer, we initiated a prospective study evaluating the roles of tumour ploidy and SPF for stomach cancer prognoses. The results are presented after a median follow-up of over 5 years.

*Received 3 June 1998*
*Revised 5 October 1998*
*Accepted 14 October 1998*

*Correspondence to:* KH Lee

### MATERIALS AND METHODS

Between November 1990 and December 1992, primary stomach cancer tissues were obtained from fresh resection specimens of 217 patients. During sampling of the tumours from the resected stomach, a pathologist examined the specimens grossly. The samples were taken from the middle portions of the bisected tumours, avoiding necrotic areas. Stromal tissues were excluded as much as possible. At the discretion of the pathologists, specimens with very small lesions were not included in the study, because sampling of such lesions may have hindered accurate pathologic examinations. In 215 cases, normal-appearing, tumour-free mucosa was also obtained from each specimen to be used as a control. Various clinical characteristics of the patients such as age, sex, duration of symptom, history of upper gastrointestinal bleeding, history of gastric outlet obstruction, history of weight loss, Karnofsky performance status, serum haemoglobin level, serum albumin level and serum creatinine level, were collected. History of bleeding and obstruction was determined to be present clinically by the presence of symptoms of haematemesis, melena, or persistent vomiting. Weight loss was determined to be present if the patient lost over 10% of his or her body weight during a 6 month period before the diagnosis of stomach cancer. Various pathologic characteristics of the tumours, such as size, location in the stomach, stage, and histologic differentiation, were also collected. The tumours were staged according to the American Joint Committee on Cancer classification (Beahrs et al, 1988). Lauren's histologic type (Lauren, 1965) was determined retrospectively by a pathologist in a blind manner.

DNA ploidy and SPF were determined by DNA flow cytometry as previously described (Lee et al, 1993). Samples were frozen rapidly in polypropylene screw-cap tubes, and stored in a freezer at – 80°C. On the day of analysis, the samples were thawed rapidly in a water bath at 37°C (Vindeløv et al, 1983*a*). Cell suspensions were prepared from the tumour and normal mucosa by mincing or scraping with a blade in RPMI 1640 (Gibco, Grand Island, NY, USA) in petri dishes, then sieving through a 30 μm nylon mesh to remove tissue fragments and cell clusters. The dissociated cells were centrifuged at 300 ***g*** for 10 min and adjusted to 1–2 × $10^6$ cells $ml^{-1}$ with RPMI 1640. Nuclear staining was done according to the methods described by Vindeløv et al (1983*b*). In brief, the stock solution was prepared by dissolving trisodium citrate dihydrate (Merck, Darmstadt, Germany), 2000 mg (3.4 mmol $l^{-1}$), Nonidet P 40 (Shell, Carrington, UK), 2000 μl (0.1% $vv^{-1}$), sperminetetrahydrochloride (Sigma, St. Louis, MO, USA), 1044 mg (1.5 mmol $l^{-1}$), and tris(hydroxymethyl)-aminomethane (Sigma), 121 mg (0.5 mmol $l^{-1}$) in distilled water to make a total volume of 2000 ml. The pH was adjusted to 7.6. Solution A was made by adding trypsin (Sigma), 15 mg in 500 ml of stock solution, and the pH was adjusted to 7.6. Solution B was made by adding trypsin inhibitor (Sigma), 250 mg, and ribonuclease A (Sigma), 50 mg, to 500 ml of stock solution, and the pH was adjusted to 7.6. For solution C, propidium iodide (Calbiochem, San Diego, CA, USA), 208 mg and sperminetetrahydrochloride, 580 mg were added to 500 ml of stock solution, and the pH was adjusted to 7.6. Solution C was protected against light with tin foil during preparation, storage, and the staining procedure. The solutions were stored in 5 ml aliquots in plastic tubes at –80°C. Before use, the solutions were thawed in a water bath at 37°C. Solutions A and B were then kept at room temperature until use. Solution C was kept in an ice bath. Solution A, 900 μl, was added to 100 μl of the cell suspension in citrate buffer and the tube was inverted to mix the contents gently. After 10 min at room temperature, during which the tube was inverted five to six times, solution B, 750 μl, was added. The solutions were again mixed by inversion of the tube, and after 10 min at room temperature, 750 μl of ice-cold solution C was added. The solutions were mixed, and the sample was filtered through a 30 μm nylon mesh into tubes wrapped in tin foil for protection of the propidium iodide against light. The samples were kept in an ice bath until analysis. The samples were run in the flow cytometer between 15 min and 3 h after the completion of staining.

The flow cytometer, FACScan (Becton Dickinson, Sunnyvale, CA, USA), was calibrated before daily use using ethanol-fixed chicken erythrocyte nuclei stained with propidium iodide. The following parameters were recorded: forward-angle light scatter, side scatter, orange-red fluorescence (FL2)-width, and FL2-area. Gating protocol was not employed. To construct each histogram, at least 20 000 events were examined after the exclusion of background, aggregates, and debris (BAD), which did not exceed 20% of the total acquired events. The G0/G1 peak of diploid cell population was set in a channel number over 50. The results were stored on disk for further analysis. The presence and the type of aneuploidy were determined according to the criteria and definition of Dressler et al (1989). DNA aneuploidy was determined to be present when two clearly-defined G0/G1 peaks were seen on a DNA histogram. DNA Index (DI) was defined as a ratio between the modal channel number for the DNA aneuploid and diploid peaks. DNA aneuploidy was further classified into five categories: simple hyperdiploidy (DI > 1.00; ≤ 1.90), near-tetraploidy (DI > 1.90; ≤ 2.20), hypodiploidy (DI < 1.00), hypertetraploidy (DI > 2.20), and multiploidy (more than 1 aneuploid peak). The SPF was obtained according to the Cellfit Software User's guide (Becton Dickinson Immunocytometry System, San Jose, CA, USA). For diploid samples, the SOBR model was used. For samples with aneuploid population, the POLY model was used. The SPF could not be obtained if the cell cycle distribution of the sample did not fit the model used. A full peak coefficient of variation for the G0/G1 peak was calculated for each sample using same software supplied by Becton Dickinson.

Frequencies of aneuploidy of tumours with various clinical characteristics and pathologic status were compared using chi-square ($\chi^2$) analysis. The SPF of tumours were compared using the Student *t*-test or analysis of variance test after log conversion. The primary end points in this study were disease-specific survival (DSS) and overall survival (OS). DSS was defined as the interval between the surgery and the death due to stomach cancer. Patients who died from causes other than stomach cancer were censored at the time of the death for the calculation of DSS. OS was defined as the interval between the surgery and the death due to any cause. Survival curves of the patients were obtained by the Kaplan-Meier method and compared using generalized Wilcoxon test. Follow-up duration of patients who were alive at the time of the analysis were considered in the calculation of the median follow-up time. For the multivariate analysis, the Cox proportional hazards regression model was used to evaluate the predictive power of various combinations of prognostic factors.

### RESULTS

### Patients

There were 148 male and 69 female patients, a total of 217 patients. The median age of the patients was 55 years (range 24–78). Of the 217 patients, grossly complete resection of tumour was possible in 197 patients. In the remaining 20 patients, gastric resection was palliative. One hundred and fifty patients received adjuvant chemotherapy (5-fluorouracil plus cisplatin in 84

*© Cancer Research Campaign 1999*

**Table 1** Patient characteristics and ploidy

| Characteristics | Frequency of aneuploidy (%) | *P* value |
|---|---|---|
| Total | 114/217 (53) | |
| Age (yr) | | 0.195 |
| ≤ 40 | 14/34 (41) | |
| 41–50 | 20/46 (43) | |
| 51–60 | 36/57 (63) | |
| 61–70 | 30/55 (55) | |
| > 70 | 14/25 (56) | |
| Sex | | 0.125 |
| Male | 83/148 (56) | |
| Female | 31/69 (45) | |
| Karnofsky performance status | | 0.299 |
| 100 | 2/7 (29) | |
| 90 | 46/79 (58) | |
| 80 | 46/85 (53) | |
| 70 or less | 18/43 (42) | |
| Tumour size (mm) | | 0.178 |
| < 40 | 16/38 (42) | |
| 40–54 | 36/57 (63) | |
| 55–79 | 32/59 (54) | |
| ≥ 80 | 30/63 (48) | |
| Tumour location | | 0.385 |
| Upper third | 8/19 (42) | |
| Middle third | 23/41 (56) | |
| Lower third | 78/143 (55) | |
| Diffuse | 4/12 (33) | |
| TNM stage | | 0.202 |
| Ia | 4/14 (29) | |
| Ib | 13/24 (54) | |
| II | 18/39 (46) | |
| IIIa | 21/40 (53) | |
| IIIb | 36/68 (53) | |
| IV | 23/32 (69) | |
| Histologic grade | | 0.001 |
| Well differentiated | 6/13 (46) | |
| Moderately well differentiated | 38/51 (75) | |
| Poorly differentiated | 64/127 (50) | |
| Undifferentiated | 4/21 (19) | |
| Histologic type (Lauren) | | 0.001 |
| Intestinal | 52/74 (70) | |
| Diffuse | 41/102 (40) | |
| Mixed | 21/40 (53) | |

**Table 2** S-Phase fraction and clinicopathologic findings

| Characteristics | *n* | Mean %S* | Range | *P* value |
|---|---|---|---|---|
| Total | 157 | 14.1 | 0–51.9 | |
| Age (yr) | | | | 0.245 |
| ≤ 40 | 28 | 13.2 | 0–49.2 | |
| 41–50 | 34 | 12.9 | 2.1–51.9 | |
| 51–60 | 38 | 15.1 | 0–45.0 | |
| 61–70 | 35 | 13.4 | 0.9–34.0 | |
| > 70 | 22 | 15.8 | 6.0–29.9 | |
| Sex | | | | 0.579 |
| Male | 101 | 13.8 | 0–45.0 | |
| Female | 56 | 14.7 | 1.2–51.9 | |
| Karnofsky performance status | | | | 0.023 |
| 100 | 6 | 6.8 | 0.9–16.8 | |
| 90 | 53 | 13.4 | 0–45.0 | |
| 80 | 65 | 13.9 | 0–51.9 | |
| 70 or less | 33 | 16.9 | 4.2–49.2 | |
| Tumour size (mm) | | | | 0.586 |
| < 40 | 31 | 16.4 | 2.6–51.9 | |
| 40–54 | 39 | 14.2 | 0.9–34.0 | |
| 55–79 | 44 | 14.4 | 0–49.2 | |
| ≥ 80 | 32 | 12.8 | 2.3–30.8 | |
| Tumour location | | | | 0.848 |
| Upper third | 14 | 11.8 | 3.3–32.4 | |
| Middle third | 31 | 14.7 | 2.1–39.3 | |
| Lower third | 103 | 14.4 | 0–51.9 | |
| Diffuse | 9 | 12.4 | 2.3–22.8 | |
| TNM stage | | | | 0.744 |
| Ia | 13 | 12.9 | 0–32.4 | |
| Ib | 17 | 15.6 | 0–51.9 | |
| II | 27 | 12.5 | 0–29.4 | |
| IIIa | 31 | 12.0 | 0.9–32.4 | |
| IIIb | 48 | 14.9 | 1.2–38.1 | |
| IV | 21 | 17.1 | 4.2–49.2 | |
| Histologic grade | | | | 0.406 |
| Well differentiated | 11 | 11.9 | 2.1–25.5 | |
| Moderately well differentiated | 32 | 12.7 | 0–32.4 | |
| Poorly differentiated | 92 | 15.3 | 0–51.9 | |
| Undifferentiated | 18 | 13.9 | 0–39.3 | |
| Histologic type (Lauren) | | | | 0.451 |
| Intestinal | 48 | 13.7 | 0–45.0 | |
| Diffuse | 80 | 14.1 | 1.2–51.9 | |
| Mixed | 28 | 14.6 | 0–49.2 | |
| Ploidy | | | | 0.087 |
| Diploid | 96 | 12.8 | 1.6–49.2 | |
| Aneuploid | 62 | 16.2 | 0–51.9 | |

*S-phase fraction patients;5-fluorouracil plus cisplatin plus levamisole in 24; oral tegafur/uracil in 41; and etoposide plus 5-fluorouracil plus leucovorin in one).

### Coefficient of variation of the G0/G1 peak

The overall mean of the coefficient of variation of the G0/G1 peak was 3.45 (range 0.01–7.20) for the samples from the normal mucosa, and 3.41 (range 0.56–6.46) for the tumours.

### Ploidy

All the 215 samples from the normal mucosa gave diploid histograms. In contrast, 114 of 217 samples (53%) from the stomach cancers gave aneuploid tracings. Among 114 aneuploid samples, there were 91 simple hyperdiploid (mean DI 1.45, range 1.06–1.90), four hypodiploid (DI 0.69, 0.88, 0.88, and 0.94 respectively), eight near-tetraploid (mean DI 2.04, range 1.94–2.18), four hypertetraploid (DI 2.41, 2.48, 2.53, and 3.10 respectively), and seven multiploid.

Moderately well differentiated tumours had a significantly higher frequency of aneuploidy compared to well differentiated or undifferentiated tumours ($P = 0.001$, Table 1). In terms of Lauren's histologic type, intestinal type stomach cancer had a significantly higher frequency of aneuploidy compared to diffuse type ($P = 0.001$, Table 1). No significant difference was observed in the frequency of aneuploidy in terms of various clinical characteristics of the patients such as age, sex, Karnofsky performance status,

© Cancer Research Campaign 1999

**Table 3** Univariate analysis: characteristics influencing disease-specific and overall survival (I)

| | Disease-specific survival | | Overall survival | |
|---|---|---|---|---|
| **Characteristics** | **Censored (%)** | ***P* value*** | **Alive (%)** | ***P* value*** |
| Age (yr) | | 0.369 | | 0.204 |
| ≤ 40 | 15/34 (44) | | 15/34 (44) | |
| 41–50 | 25/46 (54) | | 25/46 (54) | |
| 51–60 | 35/57 (61) | | 33/57 (58) | |
| 61–70 | 29/55 (53) | | 26/55 (47) | |
| > 70 | 14/25 (56) | | 11/25 (44) | |
| Sex | | 0.020 | | 0.022 |
| Male | 87/148 (59) | | 81/148 (55) | |
| Female | 31/69 (45) | | 29/69 (42) | |
| Karnofsky performance status | | 0.015 | | 0.006 |
| 100 | 7/7 (100) | | 7/7 (100) | |
| 90 | 42/79 (53) | | 41/79 (52) | |
| 80 | 48/85 (56) | | 45/85 (53) | |
| 70 or less | 21/46 (46) | | 17/46 (37) | |
| Duration of symptom (mo) | | 0.419 | | 0.473 |
| < 2 | 38/67 (57) | | 37/67 (55) | |
| 2–2.9 | 18/41 (44) | | 17/41 (41) | |
| 3–5.9 | 28/55 (51) | | 26/55 (47) | |
| ≥ 6 | 33/51 (65) | | 29/51 (57) | |
| History of bleeding | | 0.498 | | 0.210 |
| Yes | 16/34 (47) | | 12/34 (35) | |
| No | 102/183 (56) | | 98/183 (54) | |
| History of pyloric obstruction | | < 0.001 | | < 0.001 |
| Yes | 10/33 (30) | | 8/33 (24) | |
| No | 108/184 (59) | | 102/184 (55) | |
| History of weight loss | | 0.006 | | 0.004 |
| Yes | 38/86 (44) | | 33/86 (38) | |
| No | 80/131 (61) | | 77/131 (59) | |
| Serum haemoglobin level (g/dl) | | 0.101 | | 0.033 |
| < 11 | 30/55 (55) | | 27/55 (49) | |
| 11–12.5 | 25/56 (45) | | 21/56 (38) | |
| 12.6–13.9 | 28/53 (53) | | 27/53 (51) | |
| ≥ 14 | 35/53 (66) | | 35/53 (66) | |
| Serum albumin level (g/dl) | | 0.179 | | 0.092 |
| < 3.4 | 21/40 (53) | | 18/40 (45) | |
| 3.4–3.7 | 17/38 (45) | | 14/38 (37) | |
| 3.8–4.0 | 33/60 (55) | | 32/60 (53) | |
| ≥ 4.1 | 45/74 (61) | | 44/74 (59) | |
| Serum creatinine level (mg/dl) | | 0.319 | | 0.433 |
| < 0.8 | 19/43 (44) | | 18/43 (42) | |
| 0.8–0.89 | 17/28 (61) | | 16/28 (57) | |
| 0.9–0.99 | 28/57 (49) | | 27/57 (47) | |
| ≥ 1 | 53/84 (63) | | 48/84 (57) | |

*Generalized Wilcoxon test duration of symptom, history of bleeding, history of gastric outlet obstruction, history of weight loss, serum haemoglobin level, serum albumin level, and serum creatinine level. Pathologic variables such as tumour size, tumour location in the stomach, and TNM stage also failed to show significant difference in the frequency of aneuploidy (part of the data shown in Table 1).

### S-Phase fraction

The SPF was obtained in 213 of 215 samples from normal mucosa, 96 of 103 diploid tumours (93%) and 61 of 114 aneuploid tumours (54%) (157 of 217 tumours, 72%, in total). The overall mean of the SPF for the tumours was 14.1% (range 0–51.9), which is higher than that of normal mucosa (4.15%, range 0.5–31.2). The mean SPF of aneuploid tumours (16.2%, range 0–51.9) was higher than that of diploid tumours (12.8%, range 1.6–49.2), but the difference was not statistically significant ($P$ = 0.087, Table 2). The patients with poorer performance status had tumours with significantly higher SPF ($P$ = 0.023, Table 2). There was no significant difference in SPF in terms of other clinical as well as pathologic variables (part of the data shown in Table 2).

### Survival analysis

The median follow-up time of patients who were alive at the time of the analysis was 66.1 months (range 29.5–78.1). Of 217 patients, 110 patients (50.7%) were alive. Eight patients died without clinical evidence of recurrent or persistent stomach cancer and were censored for the analysis of DSS. Three patients died of postoperative complications. Each of the remaining five patients died of the following causes: sepsis after adjuvant chemotherapy, pneumonia, hepatitis B virus associated liver failure, suicidal intoxication, and trauma.

 *© Cancer Research Campaign 1999*

**Table 4** Univariate analysis: characteristics influencing disease-specific and overall survival (II)

| Characteristics | Disease-specific survival | | Overall survival | |
|---|---|---|---|---|
| | Censored (%) | *P* value* | Alive (%) | *P* value* |
| Tumour size (mm) | | 0.041 | | 0.022 |
| < 40 | 28/38 (74) | | 28/38 (74) | |
| 40–54 | 31/57 (54) | | 28/57 (49) | |
| 55–79 | 32/59 (54) | | 30/59 (51) | |
| ≥ 80 | 27/63 (43) | | 24/63 (38) | |
| Tumour location | | < 0.001 | | < 0.001 |
| Upper third | 10/19 (53) | | 9/19 (47) | |
| Middle third | 25/41 (61) | | 23/41 (56) | |
| Lower third | 81/143 (57) | | 77/143 (54) | |
| Diffuse | 1/12 (8) | | 0/12 (0) | |
| T stage | | < 0.001 | | < 0.001 |
| T1 | 20/21 (95) | | 19/21 (90) | |
| T2 | 30/33 (91) | | 29/33 (88) | |
| T3 | 57/130 (44) | | 53/130 (41) | |
| T4 | 11/33 (33) | | 9/33 (27) | |
| N stage | | < 0.001 | | < 0.001 |
| N0 | 53/65 (82) | | 51/65 (78) | |
| N1 | 38/65 (58) | | 36/65 (55) | |
| N2 | 27/87 (31) | | 23/87 (26) | |
| TNM stage | | < 0.001 | | < 0.001 |
| Ia | 14/14 (100) | | 14/14 (100) | |
| Ib | 23/24 (96) | | 22/24 (92) | |
| II | 27/39 (69) | | 25/39 (64) | |
| IIIa | 25/40 (63) | | 23/40 (58) | |
| IIIb | 21/68 (31) | | 19/68 (28) | |
| IV | 8/32 (25) | | 7/32 (22) | |
| Histologic grade | | 0.607 | | 0.597 |
| Well differentiated | 9/13 (69) | | 9/13 (69) | |
| Moderately well differentiated | 30/51 (59) | | 28/51 (55) | |
| Poorly differentiated | 66/127 (52) | | 60/127 (47) | |
| Undifferentiated | 10/21 (48) | | 10/21 (48) | |
| Histologic type (Lauren) | | 0.037 | | 0.024 |
| Intestinal | 49/74 (66) | | 47/74 (64) | |
| Diffuse | 48/102 (47) | | 43/102 (42) | |
| Mixed | 21/40 (53) | | 20/40 (50) | |
| Ploidy | | 0.410 | | 0.306 |
| Diploid | 57/103 (55) | | 54/103 (52) | |
| Aneuploid | 61/114 (54) | | 56/114 (49) | |
| S-phase fraction | | 0.007 | | 0.004 |
| < 8% | 29/49 (59) | | 28/49 (57) | |
| 8–17% | 37/59 (63) | | 35/59 (59) | |
| > 17% | 22/49 (45) | | 20/49 (40) | |

*Generalized Wilcoxon test

### Univariate analysis of prognostic factors

The results of univariate analysis of various clinicopathologic characteristics of the patients are presented in Tables 3 and 4. For the purpose of survival analysis, tumour SPFs were divided into three groups: low (less than 8%), intermediate (8–17%), and high (over 17%). The factors affecting DSS were sex ($P$ = 0.020), Karnofsky performance status ($P$ = 0.015), history of pyloric obstruction ($P$ < 0.001), history of weight loss ($P$ = 0.006), tumour size ($P$ = 0.041), tumour location in the stomach ($P$ < 0.001), T stage ($P$ < 0.001), N stage ($P$ < 0.001), TNM stage ($P$ < 0.001), Lauren's histologic type ($P$ = 0.037), and tumour SPF ($P$ = 0.007). There was no significant difference between survivals of those patients with diploid tumours and aneuploid tumours (55 vs 54% of the patients censored, $P$ = 0.410, Table 4 and Figure 1A). Those patients with tumours with an SPF over 17% had a significantly poorer DSS than those with tumours with SPFs of lower than 8% or 8–17% (45 vs 59 and 63%, $P$ = 0.007, Table 4 and Figure 1B). The factors affecting OS were similar to those affecting DSS, except that serum haemoglobin level was an additional prognostic indicator for OS ($P$ = 0.033, Table 3 and Figure 2A and 2B).

### Multivariate analysis

For the regression analyses, the following variables were considered in the variable selection process; age, sex, Karnofsky performance status, history of pyloric obstruction, history of weight loss, serum haemoglobin level, tumour size, tumour location, TNM stage, Lauren's histologic type, and tumour SPF. TNM stage remained most important indicator for DSS and OS. Tumour SPF was a significant independent variable along with tumour location (Table 5). The relative risk of dying from the disease for those patients with tumours with an SPF higher than 17% was 2.300

*© Cancer Research Campaign 1999* 

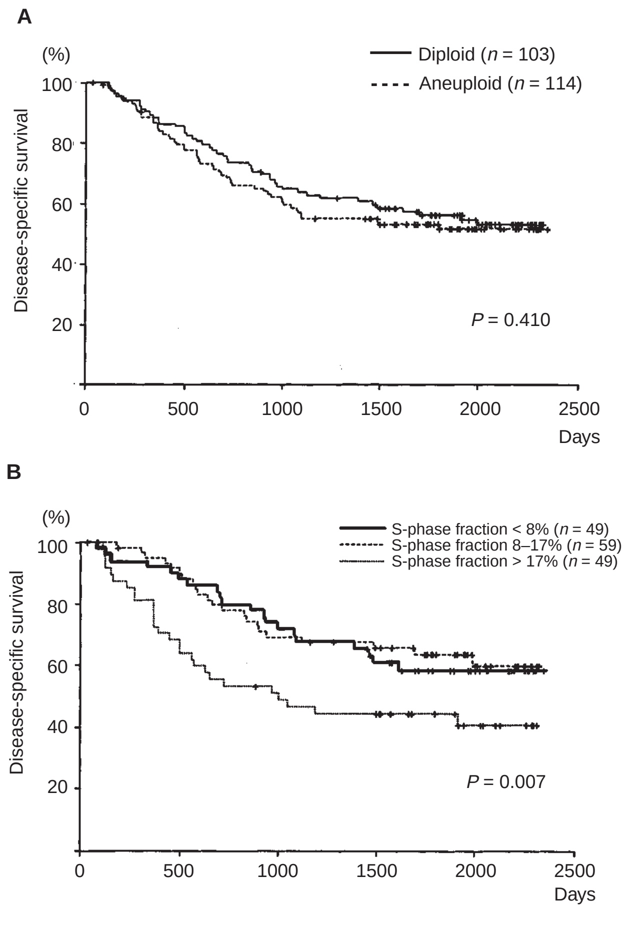

**Figure 1** Kaplan-Meier survival curves depicting disease-specific survival in patients with stomach cancer. (A) Tumour ploidy versus disease-specific survival. (B) Tumour S-phase fraction versus disease-specific survival

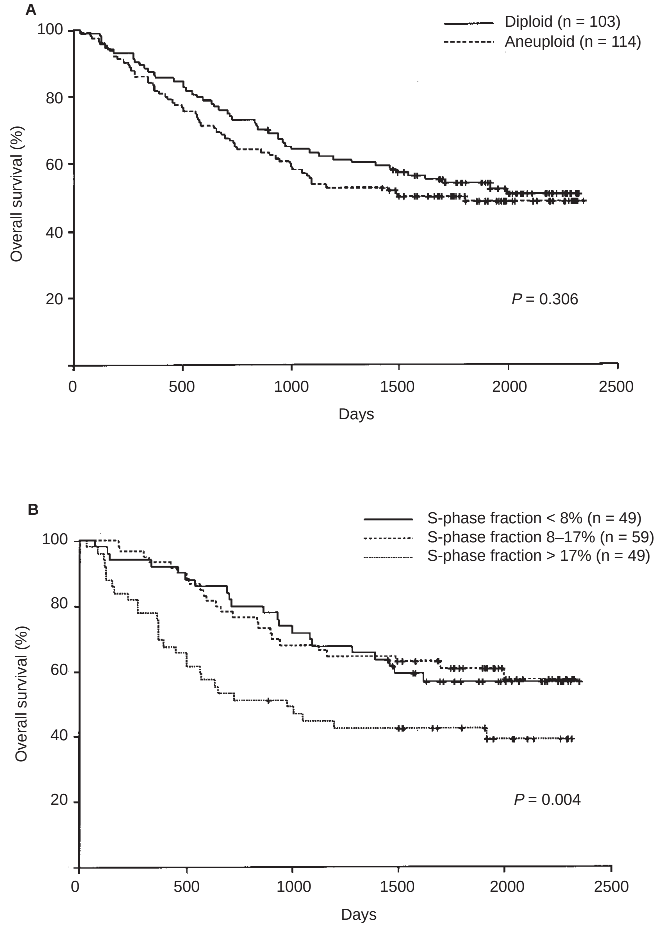

**Figure 2** Kaplan-Meier survival curves depicting overall survival in patients with stomach cancer. (A) Tumour ploidy versus overall survival. (B) Tumour S-phase fraction versus overall survival

(95% CI, 1.252–4.223) when compared to those patients with tumours with SPF lower than 8%. The relative risks of those patients with tumours with SPFs lower than 8% and 8–17% were similar (Table 5). Further analyses were done to investigate the effect of tumour SPF on DSS in each subset of patients in TNM stage. Tumour SPF had most significant influence in T3, N2, and overall stage III subsets of patients (Table 6).

### DISCUSSION

Our current study is the first in the literature investigating the prognostic value of tumour ploidy and SPF in patients with stomach cancer in a prospective manner in a large number of patients. The median follow-up duration is over 5 years. The coefficient of variation of the G0/G1 peak in our study was sufficiently low and comparable to other published data using fresh samples (Vindeløv et al, 1983*a*). It is well within 8%, which is usually recommended as an upper limit for useful SPF determinations (Shankey et al, 1993). Our findings confirm previous reports showing that the proliferative activity of tumour cells in stomach cancer is a prognostic factor (Yonemura et al, 1990; Yonemura et al, 1994; Victorzon et al, 1996). Furthermore, upon multivariate analysis, SPF was an independent prognostic indicator along with the TNM stage (Table 5). The relative risk of those patients with tumours with an SPF over 17% dying from stomach cancer was approximately twice that of patients with tumours with SPFs of lower than 8% or 8–17% (Table 5). Further analyses of patients in the subsets of the TNM stage confirmed that tumour SPF had most significant predictive power in T3, N2 and overall stage III subsets of patients, where new prognostic indicators are needed most (Table 6). The SPF could be obtained in 158 of 218 tumours (72%) in our series. When we compared two subgroups of patients with or without an obtainable tumour SPF, there was no significant difference in terms of various clinical and pathologic variables, as well as the DSS and OS of the patients (data not shown).

Tumour SPF showed significant correlation with the Karnofsky performance status of the patients ($P = 0.023$, Table 2). There was no correlation between tumour SPF and pathologic variables such as tumour size, TNM stage, and histologic grade. These findings suggest that tumour SPF is not dependent upon tumour progression or degree of histologic differentiation.

Our recent literature search retrieved 20 studies correlating tumour ploidy to patient survival in stomach cancer. Of those 20 studies, 17 studies found the presence of correlation between tumour ploidy and the survival of the patients, while the rest did not. Yonemura et al (1990) analysed the largest number of samples (493 samples) from paraffin-embedded tissues and found that tumour

© Cancer Research Campaign 1999

**Table 5** Results of multivariate analyses

| Characteristics | Relative risk (95% CI) for death: Disease-specific survival | Overall survival |
|---|---|---|
| Sex | | |
| Male | | |
| Female | 1.177 (0.705–1.9632) | 1.118 (0.684–1.828) |
| History of weight loss | | |
| Yes | | |
| No | 0.979 (0.587–1.631) | 0.892 (0.549–1.450) |
| Tumour location | | |
| Upper third | | |
| Middle third | 0.316 (0.112–0.892)* | 0.373 (0.135–1.031) |
| Lower third | 0.437 (0.172–1.114) | 0.532 (0.213–1.331) |
| Diffuse | 0.941 (0.286–3.097) | 1.230 (0.393–3.848) |
| TNM stage | | |
| I | | |
| II | 9.297 (1.092–79.122)* | 12.396 (1.517–101.266)* |
| IIIa | 14.564 (1.835–115.600)* | 17.778 (2.275–138.949)* |
| IIIb | 46.606 (6.100–356.078)* | 46.312 (6.113–350.855)* |
| IV | 68.236 (8.4797–549.162)* | 64.284 (8.076–511.689)* |
| Histologic type (Lauren) | | |
| Intestinal | | |
| Diffuse | 1.376 (0.719–2.636) | 1.313 (0.712–2.423) |
| Mixed | 1.118 (0.498–2.511) | 0.927 (0.426–2.021) |
| S-phase fraction | | |
| < 8% | | |
| 8–17% | 1.087 (0.569–2.076) | 1.158 (0.621–2.161) |
| 17% | 2.300 (1.252–4.223)* | 2.350 (1.306–4.230)* |

*$P < 0.05$

**Table 6** Disease-specific survival according to TNM stage and tumour S-phase fraction

| TNM Stage | Tumour S-phase fraction: < 8% | 8–17% | > 17% | *P* value* |
|---|---|---|---|---|
| T stage | | | | |
| T1 | 7/7 (100)** | 4/4 (100) | 6/6 (100) | – |
| T2 | 6/6 (100) | 9/11 (82) | 5/5 (100) | 0.351 |
| T3 | 14/31 (45) | 21/36 (58) | 8/31 (26) | 0.002 |
| T4 | 2/5 (40) | 3/8 (38) | 3/7 (43) | 0.309 |
| N stage | | | | |
| N0 | 15/18 (83) | 18/21 (86) | 11/12 (92) | 0.894 |
| N1 | 9/11 (82) | 12/19 (63) | 8/14 (57) | 0.347 |
| N2 | 5/20 (25) | 7/19 (37) | 3/23 (13) | 0.015 |
| TNM stage | | | | |
| I | 11/11 (100) | 9/10 (90) | 9/9 (100) | 0.368 |
| II | 6/9 (67) | 10/12 (83) | 5/6 (83) | 0.901 |
| III | 8/22 (36) | 18/32 (56) | 6/25 (24) | 0.008 |
| IV | 4/7 (57) | 0/5 (0) | 2/9 (22) | 0.274 |
| III + IV | 12/29 (41) | 18/37 (49) | 8/34 (24) | 0.002 |

*Generalized Wilcoxon test. **The numbers show the fractions of patients who are censored (%).

ploidy correlated with variables that are associated with tumour extent, such as serosal invasion, nodal spread, liver metastasis, and peritoneal metastasis, as well as patient survival. Multivariate analyses showed that tumour ploidy was an independent prognostic factor. Nanus et al (1989) analysed 50 tumour samples obtained from surgery and found that tumour ploidy correlated with disease-free survival of the patients, vertical tumour location in the stomach, and sex of the patients. There were no correlations with either depth of invasion of the tumour or nodal involvement.

In contrast to previous studies, we did not find correlation between tumour ploidy and patient survival. It is now well known that stomach cancers are heterogeneous in terms of etiology as well as epidemiology. The patient population in our study is typical of those found in high-incidence areas, i.e. the tumours are predominantly located in the distal part of the stomach. The reduction in the incidence of stomach cancer in the USA and Western Europe in the last five decades is attributable to a decline in distal lesions (Fenoglio-Preiser et al, 1996). Studies from the low-inci-

*© Cancer Research Campaign 1999* 

dence area (Nanus et al, 1989; Johnson Jr et al, 1993) showed that 42–46% of the patients in the series had stomach cancers located in the cardia, in contrast to our study where the patients with tumours located in the upper third of the stomach comprised only 9%. The difference in the pathogenesis of stomach cancers found between high- and low-incidence areas may reflect the different results between studies. In the studies of Nanus et al (1989) and Johnson Jr et al (1993), the frequency of aneuploidy of the tumours located in the cardia-gastro-oesophageal junction was found to be higher than that of tumours located in the body-antrum area of the stomach (95 vs 48%, and 39 vs 20%, respectively). We did not find such a correlation between the frequency of aneuploidy and the location of the tumour in the stomach (Table 1). When subsets of patients with stomach cancers located in the upper third or upper and middle thirds of the stomach were analysed, we did not find a significant difference in the survival of the patients according to tumour ploidy (data not shown).

The discrepancies between studies from high-incidence areas and our current study cannot be explained by the difference in the pathogenesis of stomach cancers. All of the studies from high-incidence areas which showed positive correlation between tumour ploidy and the survival of the patients were done in a retrospective manner using archived, paraffin-embedded tissues. In order to have a high level of evidence for the prognostic value of a biologic tumour marker and to evaluate the relative significance of such a role in relation to other known prognostic factors, it is of utmost importance to perform the study in a prospectively controlled manner so that there would be less chance of introduction of compounding factors. Our data is in agreement with those of Sasaki et al (1989) who performed DNA flow cytometry on 70 fresh surgical specimens, which showed a correlation between tumour ploidy and differentiation, but no correlation with patient prognosis.

In our study, tumour ploidy did not show correlation with pathologic variables that are associated with tumour progression, i.e. tumour size or TNM stage. However, tumour ploidy showed correlation with tumour grade and Lauren's histologic type. A higher frequency of aneuploidy in intestinal type stomach cancer in contrast to diffuse type (70% vs 40%, $P = 0.001$, Table 1) in our study is in agreement with a recent theory of carcinogenesis of intestinal type stomach cancer which proposed a multistep process involving metaplasia and dysplasia of gastric epithelium, along with associated genetic changes (Correa, 1992). Moderately well differentiated tumours in our study showed a significantly higher frequency of aneuploidy compared to well differentiated or undifferentiated tumours. The data, however, should be interpreted cautiously, because the frequency of aneuploidy did not change unidirectionally with the change in degree of tumour differentiation. In the study of Sasaki et al (1989), well differentiated tumours had fewer cases of aneuploidy when compared to poorly differentiated tumours, although the number of cases is small. These data need to be confirmed by further studies.

It should be noted that the patient population in our study is not representative of the population of patients with stomach cancer as a whole. Patients with very small lesions in the stomach were not included, because sampling of such lesions may hinder accurate pathologic examination. Patients with M1 lesions were also under-represented in our study, because samples were obtained from surgically resected stomach, thus excluding patients with clinically inoperable metastatic diseases. Most patients with advanced stage disease in our series received combination chemotherapy after the surgery. The impact that chemotherapy may have on the analysis of the outcome cannot be determined.

It has been reported that 33–40% of primary stomach cancers are heterogeneous in terms of tumour ploidy (Sasaki et al, 1988; De Aretxabala et al, 1989). Systematic investigation of the variation of SPF within primary stomach cancer has not been reported. Further studies are needed to determine the degree of heterogeneity of primary stomach cancer in terms of tumour SPF.

In conclusion, our study showed that tumour ploidy obtained by DNA flow cytometry did not provide prognostic information in patients with stomach cancer in a high-incidence area. On the other hand, tumour SPF was an independent prognostic factor for DSS and OS of patients with stomach cancer. Further prospectively controlled studies are warranted to confirm the prognostic value of tumour SPF in stomach cancer, especially in high-incidence areas.

### ACKNOWLEDGEMENTS

This work was supported in part by a grant (No. 99-109) from the Research Fund for the Asan Institute for Life Science.

### REFERENCES

Baretton G, Carstensen O, Schardey M and Löhrs U (1991) DNA-ploidy and survival in gastric carcinoma: a flow-cytometric study. *Virchows Archiv A Pathological Anatomy and Histopathology* **418**: 301–309

Bast RC Jr, Bates S, Bredt AB, Desch CE, Fritsche H, Fues L, Hayes DF, Kemeny NE, Kragen M, Jessup J, Locker GY, Macdonald JS, Mennel RG, Norton L, Ravdin P, Smith TJ, Taube S and Winn RJ for the American Society of Clinical Oncology (1996) Clinical practice guidelines for the use of tumour markers in breast and colorectal cancer. *J Clin Oncol* **14**: 2843–2877

Bauer KD, Bagwell B, Giaretti W, Melamed M, Zarbo RJ, Witzig TE and Rabinovitch PS (1993) Consensus review of the clinical utility of DNA flow cytometry in colorectal cancer. *Cytometry* **14**: 486–491

Beahrs OH, Henson DE, Hutter RVP and Myers MH (1988) *Manual for staging of cancer*, 3rd edn. JB Lippincott: Philadelphia

Bronzo R, Heit P, Weissman G, Kahn E and McKinley M (1989) Implications of flow cytometry in malignant conditions of the stomach. *Am J Gastroenterol* **84**: 1065–1068

Correa P (1992) Human gastric carcinogenesis: a multistep and multifactorial process – First American Cancer Society Award Lecture on cancer epidemiology and prevention. *Cancer Res* **52**: 6735–6740

D'Agnano I, D'Angelo C, Savarese A, Carlini M, Garofalo A, Bottari L, Santoro E, Giannarelli D, Vecchione A and Zupi G (1995) DNA ploidy, proliferative index, and epidermal growth factor receptor: expression and prognosis in patients with gastric cancers. *Lab Invest* **72**: 432–438

De Aretxabala X, Yonemura Y, Sugiyama K, Hirose N, Kumaki T, Fushida S, Miwa K and Miyazaki I (1989) Gastric cancer heterogeneity. *Cancer* **63**: 791–798

Dressler LG and Bartow SA (1989) DNA flow cytometry in solid tumours: practical aspects and clinical applications. *Semin Diagn Pathol* **6**: 55–82

Fenoglio-Preiser CM, Noffsinger AE, Belli J and Stemmermann GN (1996) Pathologic and phenotypic features of gastric cancer. *Semin Oncol* **23**: 292–306

Filipe MI, Rosa J, Sandey A, Imrie PR, Ormerod MG and Morris RW (1991) Is DNA ploidy and proliferative activity of prognostic value in advanced gastric carcinoma? *Hum Pathol* **22**: 373–378

Flyger HL, Christensen IJ, Thorup J, Hakansson TU and Nørgaard T (1995) DNA aneuploidy in gastric carcinoma. Flow cytometric data related to survival, location, and histopathologic findings. *Scand J Gastroenterol* **30**: 258–264

Haffty BG, Toth M, Flynn S, Fisher D and Carter D (1992) Prognostic value of DNA flow cytometry in the locally recurrent, conservatively treated breast cancer patient. *J Clin Oncol* **10**: 1839–1847

Hedley DW, Clark GM, Cornelisse CJ, Killander D, Kute T and Merkel D (1993) Consensus review of the clinical utility of DNA cytometry in carcinoma of the breast. *Cytometry* **14**: 482–485

Hermans J, Bonenkamp JJ, Boon MC, Bunt AMG, Ohyama S, Sasako M and Van de Velde CJH (1993) Adjuvant therapy after curative resection for gastric cancer: meta-analysis of randomized trials. *J Clin Oncol* **11**: 1441–1447

 *© Cancer Research Campaign 1999*

Ikeguchi M, Ohfuji S, Oka A, Tsujitani S, Maeta M and Kaibara N (1995) Aneuploidy of tumour cells in cases of gastric cancer with esophageal invasion: another indicator of poor prognosis. *J Surg Oncol* **58**: 83–90

Johnson Jr H, Belluco C, Masood S, Abou-Azama A-M, Kahn L and Wise L (1993) The value of flow cytometric analysis in patients with gastric cancer. *Arch Surg* **128**: 314–317

Kakeji Y, Maehara Y, Korenaga D, Tsujitani S, Haraguchi M, Watanabe A, Orita H and Sugimachi K (1993) Prognostic significance of tumour – host interaction in clinical gastric cancer: relationship between DNA ploidy and dendritic cell infiltration. *J Surg Oncol* **52**: 207–212

Kimura H and Yonemura Y (1991) Flow cytometric analysis of nuclear DNA content in advanced gastric cancer and its relationship with prognosis. *Cancer* **67**: 2588–2593

Korenaga D, Haraguchi M, Okamura T, Baba H, Saito A and Sugimachi K (1989) DNA ploidy and tumour invasion in human gastric cancer. Histopathologic differentiation. *Arch Surg* **124**: 314–318

Lauren P (1965) The two histological main types of gastric carcinoma: diffuse and so called intestine-type carcinoma. An attempt at a histo-clinical classification. *Acta Pathologica et Microbiologica Scandinavia* **64**: 31–49

Lee KH, Lee JS, Suh C, Ahn MJ, Kim SW, Doh BS, Min YI, Kim BS, Park KC, Lee IC, Cho YJ, Choi MG and Kim S-H (1993) DNA flow cytometry of stomach cancer. Prospective correlation with clinicopathologic findings. *Cancer* **72**: 1819–1826

Look AT, Douglass EC and Meyer WH (1988) Clinical importance of near-diploid tumour stem lines in patients with osteosarcoma of an extremity. *N Engl J Med* **318**: 1567–1572

Merkel DE, Dressler LG and McGuire WL (1987) Flow cytometry, cellular DNA content, and prognosis in human malignancy. *J Clin Oncol* **5**: 1690–1703

Nanus DM, Kelsen DP, Niedzwiecki D, Chapman D, Brennan M, Cheng E and Melamed M (1989) Flow cytometry as a predictive indicator in patients with operable gastric cancer. *J Clin Oncol* **7**: 1105–1112

Rugge M, Sonego F, Panozzo M, Baffa R, Rubio Jr J, Farinati F, Nitti D, Ninfo V and Ming S-C (1994) Pathology and ploidy in the prognosis of gastric cancer with no extranodal metastasis. *Cancer* **73**: 1127–1133

Sakusabe M, Kodama M, Sato Y, Kikuchi T and Koyama K (1996) Clinical significance of DNA ploidy pattern in stage III gastric cancer. *World J Surg* **20**: 27–31

Sarbia M, Molsberger G, Willers R, Horstmann O, Schröders C, Porschen R and Gabbert HE (1996) The prognostic significance of DNA ploidy in adenocarcinomas of the esophagogastric junction. *J Cancer Res Clin Oncol* **122**: 186–188

Sasaki K, Hashimoto T, Kawachino K and Takahashi M (1988) Intratumoral regional difference in DNA ploidy of gastrointestinal carcinomas. *Cancer* **62**: 2569–2575

Sasaki K, Takahashi M, Hashimoto T, and Kawachnino K (1989) Flow cytometric DNA measurement of gastric cancers. Clinico-pathological implication of DNA ploidy. *Path Res Pract* **184**: 561–566

Shankey TV, Rabinovitch PS, Bagwell B, Bauer KD, Duque RE, Hedley DW, Mayall BH and Wheeless L (1993) Guidelines for implementation of clinical DNA flow cytometry. *Cytometry* **14**: 472–477

Sigurdsson H, Baldetorp B, Borg A, Dalberg M, Fernö M, Killander D, Olsson H (1990) Indicators of prognosis in node-negative breast cancer. *N Engl J Med* **322**: 1045–1053

Tosi P, Leoncini L, Cintorino M, Vindigni C, Minacci C, Nuti S, Pinto E, De Stefano A and Cevenini G (1988) Flow cytometric analysis of DNA ploidy pattern from deparaffinized formalin-fixed gastric cancer tissue. *Int J Cancer* **42**: 868–871

Victorzon M, Roberts PJ, Haglund C, von Boguslawsky K and Nordling S (1996) Ki-67 immunoreactivity, ploidy and S-phase fraction as prognostic factors in patients with gastric carcinoma. *Oncology* **53**: 182–191

Vindeløv LL, Christensen IJ, Keiding N, Spang-Thomson M and Nissen NI (1983*a*) Long-term storage of samples for flow cytometric DNA analysis. *Cytometry* **3**: 317–322

Vindeløv LL, Christensen IJ andNissen NI (1983*b*) A detergent-trypsin method for the preparation of nuclei for flow cytometric DNA analysis. *Cytometry* **3**: 323–327

Wyatt JI, Quirke P, Ward DC, Clayden AD, Dixon MF, Johnston D and Bird CC (1989) Comparison of histopathological and flow cytometric parameters in prediction of prognosis in gastric cancer. *J Pathol* **158**: 195–201

Yonemura Y, Ooyama S, Sugiyama K, Kamata T, De Aretxabala X, Kimura H, Kosaka T, Yamaguchi A, Miwa K and Miyazaki I (1990) Retrospective analysis of the prognostic significance of DNA ploidy patterns and S-phase fraction in gastric carcinoma. *Cancer Res* **50**: 509–514

Yonemura Y, Fonseca L, Tsugawa K, Ninomiya I, Matsumoto H, Sugiyama K, Ohoyama S, Fushida S, Kimura H and Miyazaki I (1994) Prediction of lymph node metastasis and prognosis from the assay of the expression of proliferating cell nuclear antigen and DNA ploidy in gastric cancer. *Oncology* **51**: 251–257

*© Cancer Research Campaign 1999*
